# Redeveloping a workplace-based assessment program for physicians using Kane’s validity framework

**Published:** 2018-07-27

**Authors:** Kathryn Hodwitz, William Tays, Rhoda Reardon

**Affiliations:** 1 The College of Physicians and Surgeons of Ontario, Ontario, Canada

## Abstract

This paper describes the use of Kane’s validity framework to redevelop a workplace-based assessment program for practicing physicians administered by the College of Physicians and Surgeons of Ontario. The developmental process is presented according to the four inferences in Kane’s model. *Scoring* was addressed through the creation of specialty-specific assessment criteria and global, narrative-focused reports. *Generalization* was addressed through standardized sampling protocols and assessor training and consensus-building. *Extrapolation* was addressed through the use of real-world performance data and an external review of the scoring tools by practicing physicians. *Implications* were theoretically supported through adherence to formative assessment principles and will be assessed through an evaluation accompanying the implementation of the redeveloped program. Kane’s framework was valuable for guiding the redevelopment process and for systematically collecting validity evidence throughout to support the use of the assessment for its intended purpose. As the use of workplace-based assessment programs for physicians continues to increase, practical examples are needed of how to develop and evaluate these programs using established frameworks. The dissemination of comprehensive validity arguments is vital for sharing knowledge about the development and evaluation of WBA programs and for understanding the effects of these assessments on physician practice improvement.

## Introduction

Workplace-based assessments (WBAs) are a commonly used method of evaluating physician performance.^1^ They are routine in postgraduate training^2^ and are increasingly used with physicians in practice^3–5^ to monitor performance and promote learning and professional development through feedback.^6^ The use of WBAs for formative (learning) purposes is gaining increasing attention as public accountability and quality improvement are emphasized in healthcare.^1,7^ Hospitals and medical regulatory authorities utilize WBAs to ensure and improve physician performance^8–10^ and physicians in Canada are encouraged to pursue assessment opportunities as part of their own ongoing continuing professional development (CPD).^11–13^

As we make increasing use of WBAs to promote practice improvement, it is critical that they are explicitly developed and validated to achieve their intended effects. Validation involves the collection of evidence to support a “validity argument” that an assessment program is accomplishing its proposed purpose.^14,15^ Contemporary validity frameworks have been developed to guide this process to ensure that validity evidence (e.g., reliability of scores) is collected systematically and comprehensively.^15,16^ One such framework, developed by Kane,^14^ organizes validation as a series of inferences beginning in the “assessment world” and moving out to the “real world.” Attending to these inferences aligns the assessment with its intended use while emphasizing the effects of the assessment on those assessed.

Validity frameworks are vital to an effective validation process.^17^ However, few examples exist of how to apply validation principles and frameworks in practice.^18^ Also, validation is also an ongoing process and should be considered not only once a program is operational but while it is being developed;^14^ yet, there is a paucity of literature on how to develop effective, educationally valuable WBA programs and, to our knowledge, no previous studies on the use of validity frameworks to inform this process. Given that choices made during development can directly influence validity, it is important to consider how validity frameworks can guide WBA development, and conversely, how the developmental process can provide opportunities to collect validity evidence.

Moreover, there is considerable research on the assessment of medical residents and trainees, but far less for physicians in independent practice. Given the increasing emphasis on not only the lifelong learning of physicians but the uniqueness of learning needs at different stages of a physician’s career,^13^ it is important that research on the development of educationally-focused WBA programs extends beyond undergraduate and postgraduate environments into the practice setting.

This paper describes the use of a contemporary validity framework to redevelop a WBA program for physicians in practice administered by the College of Physicians and Surgeons of Ontario (CPSO). We will both describe the redevelopment process and present the initial validity evidence derived through this process using Kane’s validity framework.^14^ By reporting this case study, we aim to provide a practical example of how a validity framework can guide the development (or redevelopment) of a WBA program.

## Methods

### Context

As the medical regulatory authority in Ontario, Canada, the CPSO has a legislative mandate to ensure the quality and continuous improvement of licensed physicians in the province.^9^ One of the ways it fulfills this mandate is by administering a Peer Assessment program through which a randomly selected subset of physicians undergo quality improvement focused WBAs each year. Of the 30,000 physicians in active practice in Ontario, approximately 1,700 receive Peer Assessments annually.

Peer assessments are half-day WBAs conducted by trained physician assessors who practice in the same speciality and scope of practice as the assessed physician. Assessors review a sample of patient records, interview the physician, and complete a report summarizing their observations of the physician’s practice. These reports are reviewed by a Quality Assurance Committee, comprised of physicians and appointed members of the public, who decide if further follow up is needed. The majority receive satisfactory outcomes, needing no further follow up from the CPSO (93% in 2016).^19^

The Peer Assessment program has been operational since the 1980s, but underwent a significant redevelopment from 2012 to 2017 to enhance the educational value of the program. The goals were to create transparent, specialty-specific assessment criteria, improve the amount and quality of feedback provided to physicians, and systematically evaluate the acceptability and educational impact of the program for physicians. Redevelopment was led by researchers at the CPSO (the development team), in collaboration with physician assessors, utilizing best practices in program development and evaluation,^20^ principles of contemporary validity theory,^14,21^ and established criteria for high quality assessments.^22^

### Theoretical perspective

A constructivist/interpretivist perspective was assumed throughout the development and validation process which acknowledges the subjective and contextual nature of WBAs.^21,23,24^ This is in contrast to an objectivist/positivist perspective which assumes there are objective “true” scores to represent performance. WBAs measure complex, dynamic, and context-dependent behaviours occurring in unstandardized (i.e., real world) environments where assessment criteria cannot be uniformly applied.^1,21,25^ Subjectivity on the part of the assessor is not only inevitable, but critical for interpreting the nuanced aspects of performance and accounting for context.^3,21,26^ This subjectivity is not a weakness but an asset of WBAs.

As medical education moves into the “post-psychometric era” of assessment where subjectivity, expert judgement, and qualitative approaches are increasingly relied on,^21,26^ validation principles have also evolved to reflect this shift. The quality of an assessment program is now often demonstrated by its educational value, rather than solely its psychometric properties.^21,22,24^ Given the formative focus of the Peer Assessment program, we, too, put greater effort towards enhancing the educational effect of assessment for all physicians than increasing the ability to differentiate between physicians (e.g., as satisfactory or unsatisfactory).^26,27^

### Validity framework

Validation involves the articulation of an assessment’s purpose and the ongoing collection of evidence to support the use of the assessment for that purpose.^14,15^ The purpose of the Peer Assessment program was defined by the CPSO’s governing council as “to promote quality improvement by providing physicians with feedback to validate appropriate care and show opportunities for practice improvement.” The intended use of the program is to give physicians feedback about their practice and to identify if further follow up is needed (e.g., education and reassessment). We therefore collected evidence to support a validity argument that the feedback provided to physicians is useful for their professional development and that decisions regarding follow up are defensible and sound.

We used Kane’s^14^ validity framework to guide redevelopment. It organizes validity evidence according to four inferences: scoring, generalization, extrapolation, and implications (Table 1). We chose this framework for its contemporary conceptualization of validity (i.e., validity as a unified construct, supported by evidence from multiple sources) and its applicability for non-psychometric (qualitative) evidence.^15,17^ Kane’s approach requires the articulation of interpretation/use arguments, the claims underpinning each inference, followed by the collection of evidence to test these claims. The interpretation/use arguments for the Peer Assessment program are displayed in Table 1.

**Table 1:** Interpretation/Use Arguments for the Peer Assessment program

Inference	Definition	Interpretation/Use Argument
Scoring	The way in which performance is measured or scored during an assessment	Assessors will accurately and consistently provide scores (ratings and feedback) that are formatively valuable for physicians and informative for committee members.
Generalization	The degree to which the sample of performance assessed relates to performance in other situations or domains	Assessors will review a representative sample of a physician’s performance and reliably make judgements about the physician’s practice.
Extrapolation	The degree to which assessment performance reflects real-world performance	Assessment data sources reflect actual practice; assessed physicians find the assessment criteria to be acceptable; physicians agree with assessors’ interpretation of their performance.
Implications	The accuracy of interpretations and decisions resulting from an assessment and the effects of those decisions on stakeholders	Committee members have the information they need to confidently make decisions; decision making is consistent and credible; assessed physicians find the assessment to be fair, educational, and motivating for engaging in self-directed QI.

Kane’s approach emphasizes the prioritization of validity evidence based on the purpose of an assessment. Given the formative purpose of the program and our interpretivist perspective, we prioritized specific types of evidence within each inference: within scoring, we focused on how observations about performance are translated into useful feedback rather than whether the scores differentiate physicians; within generalization, we prioritized sampling and assessor training over the achievement of inter-rater reliability; within extrapolation, we emphasized the acceptability of the assessment for physicians rather than the correlation of Peer Assessments with other performance assessments; and within implications, we prioritized the educational effect of the assessment, as perceived by physicians, above quantitative outcome measures (e.g., administrative data metrics).

### Developmental approach

The development team collaborated with experienced peer assessors from a cross-section of medical disciplines throughout redevelopment. The assessment data sources (a patient record review and physician interview) remained the same but the assessment framework and tools supporting the collection of these data were redesigned. Assessor training and consensus building were emphasized throughout redevelopment, an external review process was undertaken to measure the acceptability of the new program, and an evaluation was designed to accompany the implementation of the program. These processes were undertaken as part of the CPSO’s ongoing quality improvement of its programs, thus ethical approval was not required for this work.

## Results

**Figure 1:**
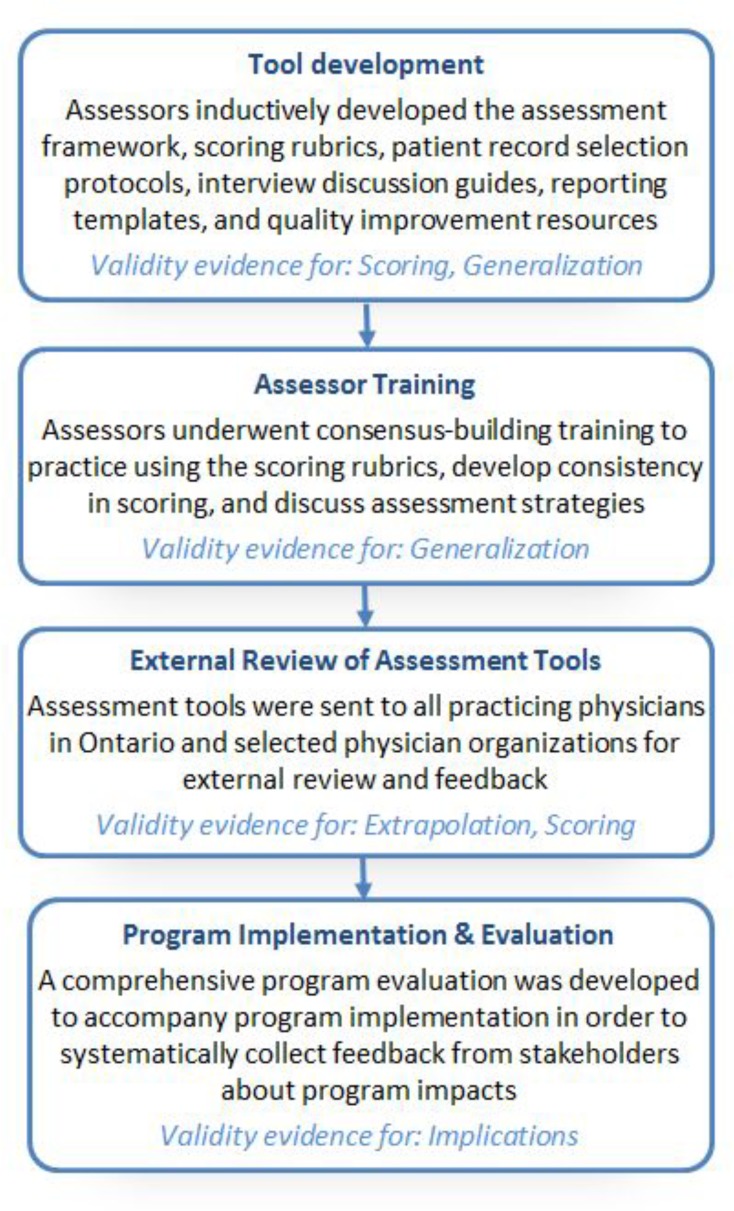
Redevelopment process and alignment with Kane’s validity framework

The redevelopment of the Peer Assessment program, and the supporting validity evidence, is presented below according to the four inferences of Kane’s validity framework. A chronological summary of the redevelopment process is displayed in Figure 1.

### Scoring

The *scoring inference* refers to the way in which observations about performance are scored. In pursuit of valid scoring, tools were developed to guide accurate and consistent performance ratings that would serve as feedback for physicians and information for committee decision makers. The assessment tools were inductively derived by peer assessors who are both content experts and the end users of the tools. A “bottom up” approach was taken wherein assessment criteria were established and agreed upon by all assessors within a specialty through iterative, consensus-building discussions facilitated by members of the development team.

An assessment framework was first developed, consisting of eight assessment domains: History, Examination, Investigation, Diagnosis, Management Plan, Medications, Follow-up and Monitoring, and Documentation for Continuity of Care. These domains were common across most specialties, but differed where appropriate (e.g., Anesthesiology developed Pre-, Intra-, and Post-procedure domains). Assessors then worked in specialty-specific groups to generate the elements of high quality care for each domain for their speciality. A three-point global rating scale was developed to accompany each domain with anchors that linked scoring with quality improvement (rather than performance quality or rank): 1) Little to no improvement needed; 2) Moderate improvement needed; and 3) Significant improvement needed. Within each domain, examples of performance for each of the three scores were populated to provide a comprehensive scoring rubric for assessor ratings (see sample in Appendix A).

Assessors also developed specialty-specific criteria for selecting patient records, discussion themes for the physician interview, reporting templates, and quality improvement resources for selected conditions or therapeutic modalities (see Table 2 for description of tools). All materials were compiled into specialty-specific handbooks and made available online to both assessors and assessed physicians.

**Table 2:** Assessment tools

Tool	Description
Patient Record Selection Protocols	Standardized criteria for how patient records are selected and reviewed
Interview Discussion Guides	Instructions on how to conduct the interview and discussion themes for promoting quality improvement
Scoring Rubrics	For each assessment domain, elements of high quality patient care and examples of care trends for each score (collectively, the assessment criteria); see Appendix A for sample
Reporting Templates	Templates for recording raw data and documenting global scores and narrative feedback
Quality Improvement Resources	Brief summaries of specific conditions, patient presentations, or therapeutic modalities, including references and resources for further information, to serve as educational material for physicians

The specialty-specific assessment criteria and improvement-focused rating scale (the scoring rubrics) align scoring with the program’s intended purpose of quality improvement (i.e., construct-aligned scoring).^17,28^ Scoring rubrics facilitate consistency in assessor ratings,^29^ and assessors’ involvement in the development of the rubrics support accurate and reliable use of the tools.^28^ Scoring rubrics also enhance the formative value of assessments by supporting the feedback process and providing explicit expectations to guide physicians’ self-directed learning.^30^

The reporting templates include global ratings and narrative comments for each assessment domain. Global ratings encourage meaningful, holistic descriptions of physicians’ practices^26^ and, compared to checklists, are more reliable and have better construct validity when used by experts.^31,32^ Narrative feedback promotes contextualized scoring and supports the formative effect of the assessment.^33,34^ The templates include headings to prompt detailed feedback^16^ and raw data (i.e., notes about each record reviewed and the physician interview) are appended to the report for transparency in how the global scores were reached.^16^

Overall, the inductive development process and extensive consensus building with assessors support accurate and reproducible scoring. The specialty-specific scoring rubrics and narrative feedback in the report support the formative utility of the scores. The usefulness of the scores for assessed physicians and committee members will be assessed through the evaluation of the program.

### Generalization

The *generalization inference* refers to how well the scores of an assessment, a subset of performance, generalize to performance across situations. The two main factors that contribute to this are adequate sampling and assessor consistency.^15^

Sampling was addressed through the development of standardized procedures for how performance data should be selected and reviewed (Table 2). Assessors select a representative sample of patient records and review the records until clear trends emerge, selecting additional records if needed to reach saturation.^27^ The physician interview clarifies trends and confirms impressions, ensuring the assessors’ report accurately represents the physician’s practice. The report includes detailed descriptions of performance with examples of how impressions were reached, as well as contextual information about the physician’s practice. These procedures for collecting and presenting narrative assessment data mirror the marks of rigour in qualitative research.^16,35,36^

Assessor consistency was facilitated through training and ongoing consensus building. While expert judgement is fundamental to performance assessments, the calibration of judgements through training is essential to ensure appropriate use of the tools and reliable, trustworthy interpretations of physician performance.^15,25–27^ In-person training sessions were conducted with all assessors, by specialty, during which assessors reviewed simulated patient records and used the scoring rubrics to independently assess the quality of care represented in the records. For each assessment domain, assessors submitted ratings anonymously and were then presented with the aggregated scores of all assessors’ ratings to view their consistency. They discussed any disagreement by sharing their perspectives on the record and then submitted a subsequent set of anonymized ratings. This process of scoring and discussion continued until an acceptable level of agreement was met, which we defined as 80% of assessors agreeing on a given rating.

Through this exercise, assessors identified areas of inconsistency in their interpretations and discussed their viewpoints until relative agreement was met. They also discussed assessment strategies and approaches; how they would use the tools to score and provide feedback to physicians. Regular assessor training sessions will be held to maintain consistency over time.

Training was also provided to CPSO staff and committee members who review and make decisions about assessment reports to ensure consistency in their processes and deliberations. The development team was present at all initial committee meetings when cases were being reviewed to provide ongoing, in-the-moment training and guidance.

The attention paid to sampling and assessor training supports the reproducibility of assessors’ judgements and the generalization of assessment results to a physician’s overall performance. Ongoing assessor and committee training will help to maintain consistency over time.

### Extrapolation

The *extrapolation inference* refers to the degree to which assessment performance reflects real-world performance. Given that patient records serve as documentation of the actual care provided to patients, a review of these data is considered representative of real performance. The inclusion of the physician interview reinforces this by ensuring accurate interpretations of the data within the context of the physician’s practice setting (e.g., the work environment or patient population).

The assessment domains and criteria were developed by physician assessors who work in a cross-section of practice environments, supporting the appropriateness of the criteria across multiple settings. An external review process of the tools was also carried out to ensure that practicing physicians deem the assessment expectations fair and appropriate. All physicians in Ontario within a given specialty were contacted by e-mail with a link to an online survey which described the Peer Assessment program and presented the assessment criteria (i.e., the scoring rubrics) for that specialty. For each assessment domain, feedback was sought about the clarity and appropriateness of the criteria and space was provided for narrative comments about suggestions for changes. In addition, relevant physician specialty organizations were contacted to provide feedback about the tools.

The external review confirmed the appropriateness of the scoring criteria (i.e., at least 80% agreement for each specialty), supporting the applicability of the assessment criteria and the acceptability of the assessment program for stakeholders.^22,37^ It also provided additional support for the *scoring inference*. The feedback collected was used to modify the tools for increased clarity (e.g., examples were added) and relevance (e.g., items were added, removed, or refined to ensure applicability to a wide range of physician practices).

The nature of the performance data sources, the inclusion of physician assessors in the development of the assessment protocols, and the external review with practicing physicians supports extrapolation theoretically. Extrapolation and acceptability will also be examined through the evaluation wherein assessed physicians will be asked if they agree with the results of their assessments.^16^

### Implications

The *implications inference* refers to the validity of the interpretations, decisions and actions resulting from an assessment as well as the effects of those actions on stakeholders. For this program, stakeholders include committee members who make decisions about assessment results and the physicians who undergo assessments.

The emphasis on rich narrative detail in the assessment reports is intended to provide committees with sufficient information to make accurate and reliable decisions about physicians’ performance. Committee training and ongoing input from the development team during committee deliberations is also intended to facilitate consistent and meaningful decisions.

Enhancing the impact of peer assessments on physicians’ learning (i.e., educational effect) and behaviour change (i.e., catalytic effect)^22^ were primary foci during redevelopment. The increased transparency in how quality care is defined and measured is intended to enhance the program’s educational effect; upon being notified of an assessment, physicians may review the assessment tools and reflect on or modify their practice prior to being assessed.^30,34^ The explicit focus on quality improvement in the revised program contributes to its intended catalytic effect: the assessment criteria define high quality patient care rather than minimum standards of practice; the assessors’ verbal and written feedback provide information about how a physician can close the gap between current performance and high quality performance, as defined in the scoring rubrics;^30,34,38^ and the Quality Improvement Resources serve as educational material for physicians, supporting self-directed QI following their assessment.

The intended implications of the program are supported theoretically. As the new program is implemented, an evaluation is being conducted of the actual implications of the program. The evaluation consists of: 1) a process evaluation to assess the impact of the program on internal stakeholders/decision-makers (i.e., assessors, program staff, committee members); and 2) an outcome evaluation to examine the impact of the program on assessed physicians. The process evaluation aims to ensure the tools are being used appropriately, the processes operate efficiently, and all operations align with the purpose of the program. This supports the outcome evaluation by increasing the ability to attribute outcomes to the activities of the program^20^ and provides information about areas for ongoing program improvements. The outcome evaluation aims to explore the potential educational and catalytic effects of the program by collecting feedback from assessed physicians approximately three months after the completion of their on-site assessment through surveys and/or interviews. Results will offer insight into the extent to which the program is achieving its intended purpose and may indicate where further development is needed to enhance its formative effects.

Evaluation mechanisms will be embedded into the program to ensure the tools remain useful and relevant. For example, assessors will be convened periodically to review the currency and relevance of the assessment criteria and regular feedback will be collected from staff, committee members, and assessed physicians about the utility, feasibility, and acceptability of the program.

## Discussion

This paper describes the redevelopment of the CPSO’s Peer Assessment program according to Kane’s validity framework. It demonstrates how a validity framework can inform the creation of a workplace-based assessment program and provides an example of development activities that correspond to the four inferences in Kane’s model. This paper also highlights how development can provide opportunities to collect initial validity evidence and identify where further evaluation efforts are needed. In this instance, *scoring* was supported through the development of specialty-specific assessment criteria and global, narrative-focused reports; *generalization* was supported through standardized sampling protocols and assessor training and consensus-building; *extrapolation* was supported through the use of real-world performance data and an external review of the scoring tools by practicing physicians; and *implications* were theoretically supported through adherence to formative assessment principles and will be tested through the process and outcome evaluations.

This evidence contributes to the overall validity argument for the assessment program, but only represents the beginning of the validation process. Validation is ongoing and will continue throughout evaluation (and future development). While it was appropriate to generate primarily “confirming” evidence during development, a critical approach will be required during the appraisal phase.^14^ During appraisal, or evaluation, evidence must be collected to critically test the assumptions underpinning each inference, with the most contentious or questionable assumptions prioritized.^14,15^ In this case, the implications for physicians will be prioritized for two reasons. First, there is limited research about the effectiveness of WBAs for quality improvement;^39–41^ implications evidence is a recognized gap in the assessment literature.^15^ Second, given that medical regulators also conduct summative assessments in response to complaints (independent of the Peer Assessment program) the regulatory context of this assessment program may detract from its intended formative effects.^42^ A critical evaluation of the implications of the Peer Assessment program will therefore be important for both the validation of the program and a broader understanding of the formative effects of WBAs, particularly those delivered by medical regulatory authorities. While attributing practice changes to any one intervention (i.e., a WBA) in a complex system such as the healthcare environment is inherently challenging, it behooves assessment administrators to evaluate the intended and unintended effects of their programs using established frameworks and to contribute to knowledge in this area.^43,44^

As the use of WBAs for formative purposes continues to increase, both the development and evaluation of these programs need to be critically examined. Given the importance placed on the lifelong learning of health professionals^13^ and the current landscape of public accountability and transparency in healthcare,^7^ it is essential that assessment programs are specifically designed to promote learning and their effects systematically evaluated and reported on. The dissemination of comprehensive validity arguments is vital for understanding of the role of WBAs in promoting practice improvement.

### Conclusion

We have provided an example of how to utilize a validity framework during the development of a WBA program. Kane’s framework was valuable for guiding the redevelopment of the Peer Assessment program and for systematically collecting validity evidence throughout this process. It brought an evaluative lens to program development and set the foundation for an effective ongoing validation process.

As the use of WBAs for formative purposes increases, further examples are needed of how to develop effective assessment programs using validity frameworks. Medical regulatory authorities and other agencies will benefit from practical examples of how to develop WBAs and collect ongoing data about the effectiveness of their mandated assessment programs. Physicians who receive these assessments will benefit from both the formative feedback and the assurance that these assessment programs are subject to critical evaluation. Members of the public whose care is enhanced through effective quality improvement initiatives are the ultimate beneficiaries of these efforts.

## Appendix A

Sample Scoring Rubric (Examination domain for Family Medicine practice)

**Table:**

**EXAMINATION:** Guided by the presenting problem, a systematic evaluation of the patient’s physical and/or mental state.	
**ELEMENTS OF QUALITY** A) **Physical examinations** were completed based on presenting complaint, with **relevant documentation** of: Pertinent positive and negative findings Physical measurements and vital signs, where appropriate Relevant descriptive information (e.g., dimensions indicating spread of cellulitis at presentation, quality of respiratory sounds; description of rash) Illustrations of conditions, where appropriate (e.g., location of rash, laceration, abdominal tenderness) B) **Mental health examinations** were completed **when indicated**, with **relevant documentation** of: Mental Status Examinations (MSEs) (e.g., mood and affect (including risk of harm to self/others), appearance, attitude, behavior, speech, thought process, thought content, perception, cognition, insight and judgment) Interplay of psychological and physiological factors C) **Standardized Measures** were completed **when indicated**, with **relevant documentation** of: Scoring flow sheets (e.g., PHQ-9, mini-mental state exam, pain scale)	
**EVALUATION CRITERIA:**	
**Score**	**Opportunities for Improvement**
1	**Little to no improvement** is needed when the trend shows that most elements of quality were evident and deficiencies, if any, were minor. Examples include: Examinations sometimes included components not relevant to the presenting complaints Mental status examinations were present but could be expanded upon
2	**Moderate improvement** is needed when the trend shows some elements of quality were lacking, but the likelihood of adverse patient outcomes was low. Examples include: Descriptions of general appearance, level of alertness, and comfort level were minimal Relevant physical measurements were not consistently present (e.g., height, weight, and BMI for preventive care and other assessments) Physical examinations were often not thorough enough to fully assess current presentations (e.g., repeated diabetic assessments with no evidence of a foot examination) Important, relevant descriptive information (e.g., dimensions indicating spread of cellulitis at presentation) was often not included Illustrated/described conditions (e.g., location of rash, laceration, abdominal tenderness) were often not included when appropriate Observations tended to be poorly described Key elements of examinations (e.g., pertinent positive and negative findings) were often not documented
3	**Significant improvement** is needed when the trend shows many elements of quality were lacking, or when patient outcomes could be adversely affected. Examples include: Pertinent vital signs (e.g., temperature and weight in child with infectious complaint) were consistently not documented Mental status examinations were often not included when relevant
